# Fabrication of Diamond Based Sensors for Use in Extreme Environments

**DOI:** 10.3390/ma8052054

**Published:** 2015-04-23

**Authors:** Gopi K. Samudrala, Samuel L. Moore, Yogesh K. Vohra

**Affiliations:** Department of Physics, University of Alabama at Birmingham, Birmingham, AL 35294, USA; E-Mails: slmoor35@uab.edu (S.L.M.); ykvohra@uab.edu (Y.K.V.)

**Keywords:** diamond anvils, chemical vapor deposition, superconductor, diamond based sensors

## Abstract

Electrical and magnetic sensors can be lithographically fabricated on top of diamond substrates and encapsulated in a protective layer of chemical vapor deposited single crystalline diamond. This process when carried out on single crystal diamond anvils employed in high pressure research is termed as designer diamond anvil fabrication. These designer diamond anvils allow researchers to study electrical and magnetic properties of materials under extreme conditions without any possibility of damaging the sensing elements. We describe a novel method for the fabrication of designer diamond anvils with the use of maskless lithography and chemical vapor deposition in this paper. This method can be utilized to produce diamond based sensors which can function in extreme environments of high pressures, high and low temperatures, corrosive and high radiation conditions. We demonstrate applicability of these diamonds under extreme environments by performing electrical resistance measurements during superconducting transition in rare earth doped iron-based compounds under high pressures to 12 GPa and low temperatures to 10 K.

## 1. Introduction

The static high pressure study on materials generally requires the use of single crystal diamonds in an opposed anvil configuration in diamond anvil cell devices. The high shear strength of diamond allows for the generation of ultra high pressure conditions at the tip of these anvils and transparency of diamond to a variety of electromagnetic radiations allows for a number of spectroscopic and diffraction measurements to be performed on materials under extreme conditions. In high pressure research, studying the electrical and magnetic properties of materials is accomplished by placing electrical probes in the sample region. These probes are in the region where shearing stress exceeds 10 GPa [1], and temperatures are very high or very low. Therefore, protecting the probes from these extreme conditions is very important to gain reliable and meaningful data. Designer diamond anvils facilitate this by encapsulating electrical probes under chemical vapor deposition grown diamond. It has been shown that designer diamond anvils can be used to study materials at extreme conditions such as megabar pressures, very low temperatures [2,3,4,5]. It has been shown previously that using a diamond anvil as a substrate, designer anvils can be produced by the use of lithography, laser pantogrpahy and CVD growth techniques [6]. These methods require the use of highly customized and expensive tools. Mask aligners need to be used for this method and physical masks tailored to each pattern to be drawn on diamond anvils need to be prepared and replaced after few uses. Such difficulties can be overcome by the use of maskless lithography. The use of maskless lithography allows us to eliminate the use of multiple systems to draw electrical circuits on diamond anvils. The maskless lithography not only allows us to fabricate designer diamond anvils but also makes it possible to fabricate other diamond based sensors such as thermocouples that can function in extreme environments. 

As mentioned earlier, several important studies have been carried out in high pressure research utilizing designer diamond anvils. The use of designer diamond anvils in diamond anvil cell allows us to use a metallic gasket for sample containment and for precise four-probe electrical resistance measurements. This is particularly helpful in observing how superconductivity changes as a function of pressure in compounds such as 1-2-2 iron (Fe)-based materials AFe_2_As_2_ (122) [A = Ba, Sr, Ca, Eu]. High pressure superconductivity in a rare-earth-doped Ca_0.86_Pr_0.14_Fe_2_As_2_ single-crystalline sample has been studied up to 12 GPa and temperatures down to 11 K using the designer diamond anvil previously [7]. These superconducting compounds are of particular interest because under pressure, superconducting transition temperature (*T*_c_) as high as ~51 K at 1.9 GPa has been observed, presenting the highest *T*_c_ reported in the intermetallic class of 1-2-2 iron-based superconductors. In this paper we report results from our study on a rare-earth doped iron-based superconductor Ca_0.9_Pr_0.1_Fe_2_As_2_ using a newly fabricated designer diamond anvil.

## 2. Experimental Section

**Figure 1:**
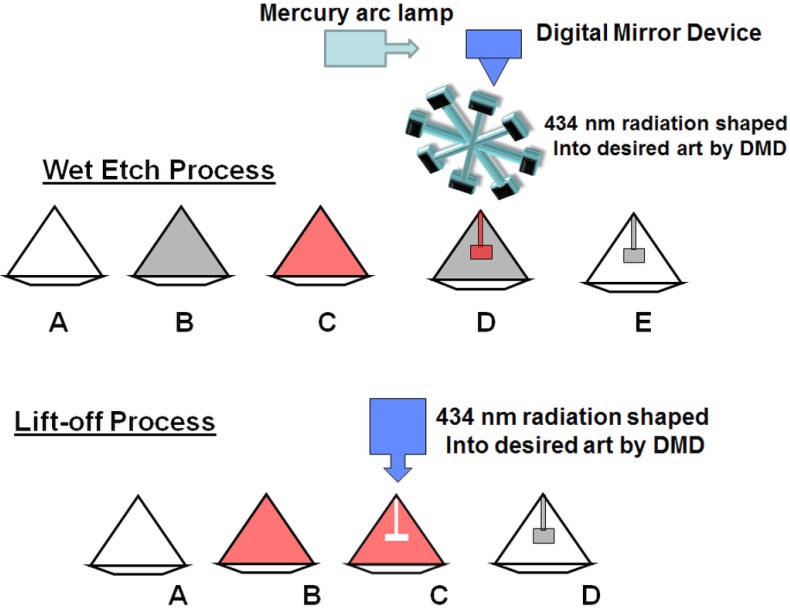
The top row shows the steps involved in fabricating a designer diamond through the wet etch process. The bottom row shows the steps involved in fabricating designer diamond anvils through the lift-off process.

Type-Ia and Type-IIa gem quality diamonds are utilized to fabricate the designer diamond anvils. High resolution and highly customizable circuit patterns have been imprinted onto single crystal diamond (SCD) substrate anvil surfaces prior to being entirely encapsulated in SCD utilizing the following instrumentation: DC-sputter deposition, maskless lithography and microwave plasma chemical vapor deposition (MPCVD). The DC-sputter deposition system is AJA International Inc.’s (Scituate, MA, USA) Orion sputtering system with a sputter down configuration. The maskless lithography system is SF-100 Xcel system from Intelligent Micro Patterning LLC (St. Petersburg, FL, USA). The maskless system allows us to draw patterns on samples with extreme topography such as diamond anvils which have angles between surfaces ranging from 7° to 45°. This is very important because we need to extend electrical probes down onto facets of diamond anvils as shown in Figure 1. The MPCVD system is a custom built system at University of Alabama at Birmingham. The anvil geometries utilized consist of original culet sizes ranging from 10 to 600 microns, with bevel angles (angle between the culet and facet) ranging from 7° to 50°. Two lithographic process techniques have been used to achieve the desired results and are outlined below:

Method 1: The top row (TR) of graphics in Figure 1 outline the steps of obtaining encapsulated metallic circuits on diamond anvils with the use of positive (or negative) tone resist and wet etching. In this process, the bare diamond anvil substrate (TR-A) has been etched in a gentle RF-Plasma in the sputter deposition chamber prior to sputter coating. During the sputtering process, an RF bias has been maintained to ensure we deposit good quality metal film on our substrates. The substrate then has a uniform tungsten metal layer sputter deposited onto its surface so that the entire substrate is coated with a metallic layer (TR-B). The thickness of the metallic coating is in the range of 0.1–2 microns. After the metal has been sputter coated, photoresist (both positive tone and negative tone resists have been utilized to achieve the final result) is applied to the diamond using a spin coater with angular velocity ranging from 1000 to 12000 rotations per minute (rpm). The angular velocity is varied according to the anvil geometry in order to apply a uniform resist coating (TR-C). The resist has been processed according to manufacturer’s recommendation by baking it at suitable temperature. The maskless photolithography instrument is then implemented to expose the substrate in very specific regions with visible light in the 360–450 nm range in a very high resolution pattern (micron scale resolution is achieved) via the digital micro mirror device (DMD) contained as an internal component of the maskless lithographic instrument (TR-D). Depending on the tone of the resist (positive resist becomes soluble when exposed to radiation, whereas negative resist becomes insoluble), the artwork loaded into the lithographic system software is designed specifically to meet the final circuit dimension specifications. The exposed resist is then placed in a developer solution and a pattern is rendered in the resist layer. The resist that remains is essentially a protective layer for the underlying tungsten film during the next phase of wet etching the substrate in a weakly acidic tungsten etchant. Once the etchant has completely dissolved the excess tungsten in the base layer, the substrate is removed from the solution and the photoresist layer is dissolved in a solvent. The result is a diamond anvil with a metallic pattern drawn on it (TR-E). The diamond anvil is then transferred into a 1.2kW MPCVD chamber and SCD is grown at temperatures ranging from 800 to 1300 °C with a CH_4_/H_2_ ratio of 1%–10%. This results in the growth of CVD diamond with a thickness of 10–70 microns. The anvil is then polished until electrical contacts on the culet surface are exposed and the diagnostic contact pads on the facets are all exposed, facilitating the connection of external laboratory equipment.

Method 2: The bottom row (BR) of Figure 1 outlines the liftoff process. Lift off process is essentially the reverse process of the wet etch procedure previously described. In the lift off process the same instrumentation, materials and process parameters outlined in the wet-etch method are used to perform the fabrication process. However, in this method resist coating and all lithographic process steps occur on the bare SCD substrate prior to sputter deposition (BR-A,B). As a result, once the lithographic process is completed the diamond surface regions in which metal will be deposited to render the final circuit pattern are exposed to incident tungsten atoms (BR-C). During sputter deposition tungsten particles coat these regions and adhere to the diamond substrate surface. After sputter deposition, the resist layer is stripped from the diamond anvil, and the excess tungsten deposited on top of the resist layer will be removed and only the final circuit pattern remains (BR-D). Resolution enhancement has been achieved by depositing an interstitial under layer of resist prior to applying an imaging resist layer. In this bi-layer method the interstitial layer (base layer of resist deposited directly onto the diamond) has a slightly higher dissolution rate than the imaging resist that is deposited on top of it, this results in an “overhang” effect during the development phase which leads to improved resolution as sputter deposited material is less likely to delaminate from the diamond surface due to attachment of tungsten to the resist coating during the resist stripping phase. Once sputter deposition and resist stripping is complete, the anvil undergoes the same steps as in the encapsulating MPCVD and polishing phase described in method 1.

## 3. Results and Discussion

**Figure 2:**
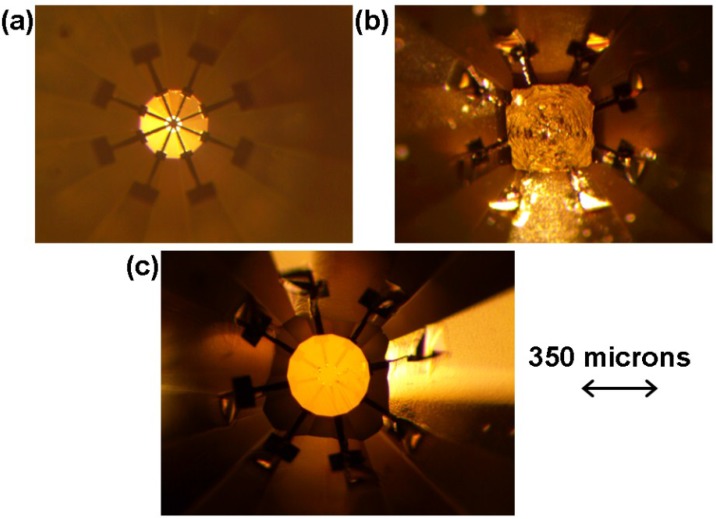
(**a**) Eight electrical probes lithographed onto diamond anvil; (**b**) CVD diamond grown on top of eight probe pattern; (**c**) A fully fabricated designer diamond anvil with a culet size of 370 microns with a probe circle diameter of 85 microns.

A diamond anvil with a central flat size of 70 microns in diameter, beveled at 7.5 degrees to a culet size of 350 microns in diameter has been chosen as base substrate for the fabrication of a designer diamond anvil. A tungsten film of 0.5 microns thick has been sputter deposited onto this diamond anvil. This diamond was then coated with Shipley 1827 positive photoresist. Utilizing an eight electrical probe design graphic file as an input for the maskless lithography system, we have removed photoresist from unwanted areas. The first step in the fabrication of this designer diamond was completed after developing the photoresist, and wet etching step to remove tungsten from unwanted areas. Figure 2a shows the resulting 8 probe pattern made of tungsten metal on the diamond anvil substrate. The width of metallic probes is 10 microns and their thickness is 0.5 microns. Figure 2b shows the CVD diamond grown on top the eight probe pattern to encapsulate it. The fabrication of the designer diamond anvil was completed by polishing the CVD diamond layer and exposing the probes in the sample region. Figure 2c shows a fully finished designer diamond anvil with eight electrical probes. The final culet size of this designer diamond anvil after polishing is 370 microns. The diameter of the circle of probes that have been exposed is 85 microns. These dimensions allow for researchers to include high volume of material in the high pressure research experiments. Designer diamond anvils with this geometry have been utilized in studying the electrical and magnetic properties of materials such as rare earth elements gadolinium, dysprosium [4,5].

The 1-2-2 Fe-based materials AFe_2_As_2_ (122) [A = Ba, Sr, Ca, Eu] have ThCr_2_Si_2_ type tetragonal crystal structure at ambient conditions and are among the widely studied class of Fe-based superconductors. These compounds exhibit their superconductivity upon application of high pressure or chemical doping once the magnetic ordering is suppressed [8]. The superconducting transition temperature (T_c_) in the 1-2-2 family has been limited to low temperatures with the maximum being 38 K observed in hole-doped Ba_1-x_K_x_Fe_2_As_2_ [9]. However, unusually high superconducting transition temperature characterized by a mysterious small volume fraction which superconducts up to 45–49 K, has been recently realized in single crystalline rare-earth (RE) doped Ca_1-x_Pr_x_Fe_2_As_2_ [10,11,12]. It is even more surprising to note that the superconducting state for Ca_1-x_Pr_x_Fe_2_As_2_ with an onset T_C_ of 49 K is non-bulk or filamentary, highly anisotropic, and displays a Josephson coupling characteristic and double resistive transition as evidenced from the resistive and magnetic results of single crystalline sample at ambient pressure [10,11]. Presently, limited high pressures studies have been reported [12] on these compounds and it is not well known whether high pressure and electron doping play a similar role in optimizing the superconducting properties of the RE doped CaFe_2_As_2_ material.

**Figure 3:**
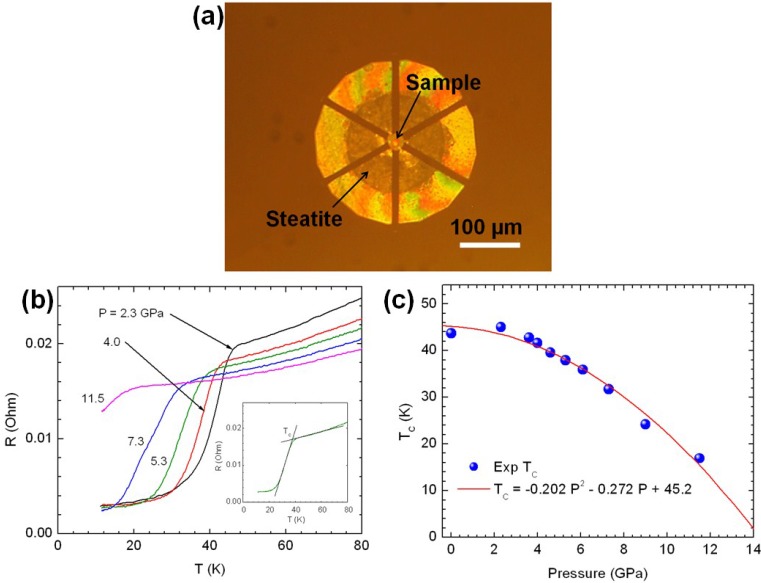
(**a**) Ca_0.9_Pr_0.1_Fe_2_As_2_ sample loaded in a diamond anvil cell along with steatite pressure medium, and ruby for pressure calibration; (**b**) Electrical resistance data collected with a designer diamond anvil. The insert shows our method of determining the onset of superconductivity (*T_c_*); (**c**) *T_c_* as a function of pressure for Ca_0.9_Pr_0.1_Fe_2_As_2_.

Figure 3a shows the iron-based superconducting sample in a diamond anvil cell sample chamber assembly. The sample chamber in a stainless steel gasket is 120 microns in diameter and has an initial thickness of 70 microns and contains Ca_0.9_Pr_0.1_Fe_2_As_2_ sample along with a ruby pressure marker surrounded by an electrically insulating pressure medium steatite. The pressure was continuously monitored utilizing the spectral location of Ruby R_1_ fluorescence emission at high pressures and low temperatures [7]. Figure 3b shows the four probe electrical resistance as a function of temperature at various pressures. The onset temperature of superconductivity (*T_c_*) is marked by a sharp downturn in electrical resistance and the insert in Figure 3b illustrates our methodology for the determination of *T_c_*. Figure 3c shows a plot of *T_c_* as a function of pressure and illustrates a gradual decrease of T_c_ with increasing pressure and can be fitted to the following quadratic equation.

T_c_ (in Kelvin) = 45.2 − 0.272 P − 0.202 P^2^ (P is in GPa units) (1)

The present study shows that the superconducting transition temperature (T_c_) in Pr-doped CaFe_2_As_2_ is a monotonically decreasing function of pressure for a 10% doping level and the highest value of T_c_ of 45 K is observed at ambient pressure. The rare-earth doped CaFe_2_As_2_ materials continue to show the highest superconducting transition temperatures amongst the family of 1-2-2 iron-based superconductors [7,11,12,13]. In addition, a two-step superconductivity behavior with two different transition temperatures observed for 14% doping level in Pr-doped CaFe_2_As_2_ [7] is not reproduced in the 10% sample. This points to the need for *simultaneous* measurements of electrical properties (or T_c_) and crystal structures on the same sample to correlate observed superconducting behavior and the crystalline phases at a given temperature and pressure. Such *simultaneous* measurements of superconductivity and crystal structures at high-pressure and low-temperatures are possible with the designer diamonds fabricated in this study. 

We have described a novel method for fabrication of designer diamond anvils utilizing a combination of maskless lithography, sputtering and MPCVD techniques. However, the versatility of the maskless system allows us to fabricate a variety of other diamond based sensors. One sensor that is currently under development is a thermocouple that can function in any extreme environment. Thin-film thermocouples have been fabricated in earlier studies on substrates such as ceramics and superalloys [14]. These materials have their limitations as thermocouple metals exposed to extreme environments will undergo oxidations and will suffer mechanical damage compromising their integrity. Such hazards can be avoided by sputter deposition of thermocouples on diamond substrates and encapsulating them within a single crystalline CVD diamond layer. The high thermal conductivity; chemical, radiation inertness of diamond make it an ideal candidate as a base material for building thermocouples. By utilizing sputtering, maskless lithography; thermocouple alloys can be sputter deposited on diamond substrates. Patterns made of thermocouple alloys with features down to 5 microns in width and 0.5 microns thickness can be fabricated on diamond substrates utilizing the methods described in this paper. These alloys can be encapsulated under CVD grown diamond and can be exposed in strategic locations to make contact with laboratory equipment.

## 4. Conclusions

In summary, a versatile and easy to use method for fabricating designer diamond anvils using maskless lithography is developed. The methodology demonstrated in this paper not only allows for the fabrication of sensors for high pressure research, but has the potential to develop other diamond based sensors such as thermocouples which can function in extreme environments. As an application of this technology, superconducting behavior of a rare-earth doped iron-based compound has been studied to high pressures of 12 GPa and temperatures down to 10 K. The materials fabrication processes described in this paper can extend the use of diamond-based sensors in a wide range of extreme conditions of pressures, temperatures, radiation and chemical corrosive environments. 
